# Kernel generalized least squares regression for network-structured data

**DOI:** 10.1371/journal.pone.0324087

**Published:** 2025-05-30

**Authors:** Edward Antonian, Gareth W. Peters, Michael Chantler

**Affiliations:** 1 School of Mathematics and Computer Science, Heriot-Watt University, Edinburgh, United Kingdom; 2 Department of Statistics and Applied Probability, University of California Santa Barbara, Santa Barbara, California, United States of America; Michigan State University, UNITED STATES OF AMERICA

## Abstract

In this paper, we study a class of non-parametric regression models for predicting graph signals $\left\{{𝐲}_{t}\right\}$ as a function of explanatory variables $\left\{{𝐱}_{t}\right\}$. Recently, Kernel Graph Regression (KGR) and Gaussian Processes over Graph (GPoG) have emerged as promising techniques for this task. The goal of this paper is to examine several extensions to KGR/GPoG, with the aim of generalising them a wider variety of data scenarios. The first extension we consider is the case of graph signals that have only been partially recorded, meaning a subset of their elements is missing at observation time. Next, we examine the statistical effect of correlated prediction error and propose a method for Generalized Least Squares (GLS) on graphs. In particular, we examine Autoregressive AR(1) vector autoregressive processes, which are commonly found in time-series applications. Finally, we use the Laplace approximation to determine a lower bound for the out-of-sample prediction error and derive a scalable expression for the marginal variance of each prediction. These methods are tested on both real and synthetic data, with the former taken from a network of air quality monitoring stations across California. We find evidence that the generalised GLS-KGR algorithm is well-suited to such time-series applications, outperforming several standard techniques on this dataset.

## 1 Introduction

Graphs have proven to be a useful way of describing complex datasets due to their ability to capture general relational information between entities [1]. Knowledge about pairwise relationships between data points can, for example, help enhance regularization, improve computational efficiency and robustness, or better exploit unlabelled data [2,3]. Successful applications have included social networks [4], brain imaging [5], biomolecular systems [6], sensor networks [7], image and point cloud processing [8] and chemical structure [9] to name a few.

The Graph Signal Processing (GSP) community focuses in particular on generalisations of traditional signal processing techniques on regular domains. A toolbox of analogous graph-based techniques has been built by translating concepts such as convolution and spectral decomposition into the graph domain. These have been applied to a diverse range of problems including signal reconstruction [10], graph learning [11], denoising [12] and regression [13].

Graph-based regression specifically has received some attention recently in the form of kernel regression and Gaussian processes [13–16]. In this context, we are interested in constructing a function that maps input features to an output space, which is interpreted as a graph signal. This opens interesting avenues for enhanced regularisation using GSP tools and offers novel ways of defining kernel functions.

In this paper, we examine Kernel Graph Regression (KGR) and the closely related topic of Gaussian Processes over Graph (GPoG) from the perspective of Graph Signal Reconstruction (GSR). This framework allows signal prediction at unobserved test nodes as well as test times. Next, we extend this by developing a method for Generalized Least Squares (GLS) regression on graphs. This has particular relevance for the statistical analysis of time-series data where a graph signal is sampled at regular intervals, as it allows, for example, node-level error autocorrelation to be incorporated. Such scenarios are encountered in the analysis of many common graph applications such as sensor networks and financial time series. Finally, we use the Laplace approximation to calculate a lower bound for the out-of-sample prediction uncertainty and derive a scalable expression for the marginal uncertainty at each node.

### 1.1 Problem overview and scope

The goal of supervised learning and regression is to estimate an optimal function that maps an input space to an output space, given a set of training examples $\{({𝐱}_{t},{𝐲}_{t}){\}}_{t=1}^{T^{\prime }}$. It will be assumed that there is a static graph 𝒢={𝒩,ℰ,𝐀} with vertex set 𝒩 with *N* vertices (nodes), edge set ℰ and adjacency matrix 𝐀. In this paper we are concerned specifically with real-valued vector regression inputs (time series covariates) and outputs (time series responses), that is, ${𝐱}_{t}\in {\mathbb{R}}^{M}$ and ${𝐲}_{t}\in {\mathbb{R}}^{dN_{t}^{\prime }}$, for *d*-dim observation vector over a subset ${𝒩}_{t}^{\prime }\subseteq 𝒩$ of $N^{\prime }$ vertices out of a total of *N* vertices at time *t*. Clearly, it is assumed that ${𝐲}_{t}$ is a partially observed graph signal, meaning the elements can be interpreted as being observed at time *t* on a subset ${𝒩}_{t}^{\prime }\subseteq 𝒩$ of the full node-set $𝒩=\left\{v_{1},\ldots ,v_{N}\right\}$.

In this setup, the *M* dimensional covariate time series ${𝐱}_{t}$ may be exogenous to the graph vertices (not observed at any of the 𝒩 graph vertices) or they may be endogenous to the graph vertices (observed at a subset or all of the graph vertices). The methodology developed in this work admits both possible setups, but the focus in this work will be primarily on the former case of exogeneity of the regression covariate time series and this case will be considered unless otherwise stated.

The $dN^{\prime }$ dimensional response time series vectors ${𝐲}_{t}$ of the graph regression will be assumed to be observed, ideally at each time point t∈{1,…,T}, such that the first *d* coordinates ${𝐲}_{i,t}$ for i∈{1,…,d} corresponds to the *d*-dimensional observation sub-vector of ${𝐲}_{t}$ that were observed at vertex $v_{1}\in {𝒩}_{t}^{\prime }\subseteq 𝒩$ and analogously coordinates ${𝐲}_{i,t}$ for i∈{(j−1)d+i,…,(j−1)d+d} corresponds to the *d*-dimensional observation sub-vector of ${𝐲}_{t}$ for vertex $v_{j}\in {𝒩}_{t}^{\prime }\subseteq 𝒩$.

In the case that the cardinality of vertex set ${𝒩}_{t}^{\prime }$ is less than that of the complete graph vertex set 𝒩 i.e. $\left|{𝒩}_{t}^{\prime }|<|𝒩\right|$, the formulation will accommodate missingness of observations at some vertices in the setup, allowing for a subset of the vertices, ${𝒩}_{t}^{\prime }$, to have complete missingness of the observation vectors per time instant. In the case that $\left|{𝒩}_{t}^{\prime }|=|𝒩\right|$ one may have full or partial observations all d-dim observation sub-vector coordinates per vertex ${𝐲}_{t}$ at all vertices in 𝒩.

This gives a general pattern of missingness where single observation coordinates of missingness may occur at a given vertex or an entire observation vector may be missing at a vertex at any instant of time. To capture this general pattern of missingness, a general sensing matrix $S\in {\left[0,1\right]}^{dN\times T}$ will be utilised throughout, where *S*_*i*,*t*_ takes value 1 if the *i*-th coordinate of ${𝐘}_{t}$ is observed at time *t* and value 0 if it is missing at time *t*. Without loss of generality, in latter sections, we will set *d* = 1 and we will decompose *S* into two sub-matrices $S_{N}\in {\left[0,1\right]}^{N^{\prime }\times N}$ and $S_{T}\in {\left[0,1\right]}^{T^{\prime }\times T}$.

The graph itself is assumed to be static in time with a known adjacency matrix $𝐀\in {\mathbb{R}}^{N\times N}$, for a graph with *N* vertices. There will be an estimation challenge to estimate the graph signal reconstruction and regression models over *T* time points based on a potentially partially observed set of responses over *N* vertices at $T^{\prime }\leq T$ time points. The types of missingness that can be considered in this setup is quiet general and this aspect will be explained with illustrations in latter sections of the paper.

In this formulation, the input features ${𝐱}_{t}$ are ‘global’ in the sense that individual elements are not necessarily associated with any particular node in the output space. For example, if the graph represents a network of corporations, and the graph signal is their quarterly revenue growth, the features could represent global factors such as inflation, GDP etc, *as well as* firm-specific data such as the number of employees at a particular company. This is the most general formulation and allows for maximum flexibility in problem specification. Additional constraints explicitly linking elements from one space to the other are possible but not considered in this paper. This approach is shared by other recent work such as [13,17].

Throughout this paper we take a Bayesian perspective, using graph filters to construct spectral priors that can be used to make statements about the expected profile of a graph signal. Using graph filters in this way shares aesthetic and practical features with the graph kernel framework described in [18,19] and elsewhere. Graph filters as opposed to kernels, however, provide a more direct Bayesian interpretation which is useful in this context.

As mentioned, in this work, the graph adjacency matrix 𝐀 is assumed to be known a priori. In many applications such as social or sensor networks, this is a reasonable assumption, as a graph may be self-evident or easy to construct. For other applications, such as financial or biological networks, the dependency structure may be more opaque. In such scenarios, we refer the reader to the large body of work on graph learning [20–23].

### 1.2 Contributions

This paper contributes a statistical exploration of Kernel Graph Regression and Gaussian Processes over Graphs in the context of graph signal reconstruction. A primary aim is to incorporate the possibility of error correlation, which is of practical concern in many applications. We pay particular attention to time series modelling and node-level autocorrelation and describe an algorithm for determining function and model parameters in the presence of autocorrelated noise. We also focus on deriving mathematical expressions which lend themselves to practical computation for large-scale problems and use Bayesian reasoning to provide a lower bound for the model parameter uncertainty. These contributions are clarified below.

#### 1.2.1 Incorporating partially observed graph signals

Prior work on KGR and GPoG has assumed that the signal from all nodes under consideration is fully observed throughout training [24], [25] and [26]. We introduce a modification that incorporates ideas from graph signal reconstruction to allow a constant subset of nodes to remain unobserved at train time and use the topology of the graph to make smooth predictions at these points.

#### 1.2.2 Incorporating correlation prediction error via Generalised Least Squares (GLS)

We relax the statistical assumption that all prediction errors should be independent and identically distributed, allowing for the possibility of cross-correlation amongst nodes, and autocorrelation over time. This creates a broader class of models with rich statistical properties. In particular, we consider the scenario where Autoregressive AR(1) autocorrelation may exist at each node which has relevance in many time-series applications.

#### 1.2.3 Strict enforcement of worst-case *O*(*N*^3^  +  *T*^3^) complexity

Graph regression problems are naturally expressed in terms of matrix operations of (N,N) ⊗ (T,T) Kronecker products i.e in terms of a matrix operations on an *NT* × *NT* matrix of elements involving a high dimensional inverse. This is problematic for all but the smallest of problems as memory and computational costs escalate quickly. This work contributes an analysis of the complexity of the methods and ensures that all final mathematical expressions are given in terms of worst-case $O(N^{3}+T^{3})$ operations, ensuring medium to large problems remain tractable.

#### 1.2.4 A Laplace approximation for parameter uncertainty

We use the Laplace approximation to estimate the full posterior and derive a tractable expression for the marginal variance. This can be used to provide a lower bound for the uncertainty over out-of-sample model predictions.

### 1.3 Paper organisation

Section 2 gives a brief introduction to some fundamental GSP concepts such as the Graph Fourier Transform (GFT) and graph filters. In section 3 we revisit the problem of Kernel Graph Regression (KGR) from a Bayesian perspective and incorporate the concept of partially observed graph signals. Section 4 builds on this, introducing the GLS model and outlining an iterative algorithm for estimating the prediction error, focusing particularly on an autoregressive model. Here, we also run several experiements on synthetic data to provide intuition for the hyperparameters this algorithm relies on. Finally, section 5 uses the Laplace approximation to derive a tractable lower bound for the marginal prediction uncertainty. Finally, section 6 analyses these methods on a real dataset concerning the prediction of pollutant levels across a network of monitoring stations in California.

### 1.4 Notation

Effort is made to adhere to the following variable naming conventions throughout this paper, see Table 1.

**Table 1:** The notational conventions used in this paper.

Object	Description	Example
Integer variable	Lower case Latin	*n*,*t*,*m*
Integer constant	Upper case Latin	*N*,*T*,*M*
Scalar variable	Lower case Greek	α,β,ξ
Vector	Lower case bold Latin	𝐚,𝐱,𝐲
Vector element	Lower case sub lower case Latin	$\lambda_{t},f_{j},y_{i}$
Matrix	Upper case bold Latin or Greek	$𝐋,\Sigma_{N},\Lambda_{K}$
Matrix element	Matrix sub double lower case Latin	${𝐉}_{nt}$
Matrix column	Matrix sub lower case Latin	${𝐉}_{n}$
Transpose	Symbol raised to ⊤	${𝐗}^{⊤}$
Identity matrix	Capital 𝐈 sub dimension	${𝐈}_{N}$
Optimal value	Symbol raised to ⋆	${𝐅}^{⋆}$

## 2. Preliminaries

### 2.1 Graphs and the GFT

Consider a weighted, undirected graph 𝒢=(𝒱,ℰ,𝐀) made up of *N* vertices $𝒱=\left\{v_{1},\ldots ,v_{N}\right\}$ and edge set $ℰ=\left\{e_{i,j}=(i,j):v_{i}\sim v_{j}\right\}$ connecting vertex pairs. $𝐀\in {\mathbb{R}}^{N\times N}$ is the weighted adjacency matrix, where ${𝐀}_{ij}={𝐀}_{ji}$ represents the strength of interaction between nodes *i* and *j*. (i,j)∈ℰ implies ${𝐀}_{ij}>0$ and (i,j)∉ℰ implies ${𝐀}_{ij}=0$. For graph 𝒢 one may define the graph Laplacian matrix as 𝐋=𝐃 − 𝐀 where 𝐃 is the diagonal degree matrix; ${𝐃}_{ii}$ being the *i*th row sum (or column sum) of 𝐀. One key property of the graph Laplacian is that it can be used to measure the smoothness of a signal $𝐟\in {\mathbb{R}}^{N}$ with respect to the graph.

$${𝐟}^{⊤}𝐋𝐟=\sum_{(i,j)\in ℰ}{𝐀}_{ij}(f_{i}-f_{j}{)}^{2}\geq 0$$ (1)

As visible, the quadratic product ${𝐟}^{⊤}𝐋𝐟$ measures the square difference in signal value between connected nodes, weighted by the respective adjacency matrix entry. In this way, it can be considered a measure of ‘roughness’ of the signal **f**, with smaller values indicating a smoother signal, achieving a minimum of zero when the function is constant on each disconnected subgraph [27]. It can be useful to view the Laplacian in terms of its eigendecomposition, $𝐋=𝐔\Lambda_{L}{𝐔}^{⊤}$, where the columns of the orthogonal matrix 𝐔 are the normalised eigenvectors of 𝐋, forming an orthonormal spanning set. (The eigenvalues are typically ordered such that $\lambda_{1}^{L}\leq \lambda_{2}^{L}\leq ...\leq \lambda_{N}^{L}$). Any graph signal can be expressed as a linear combination of the eigenvectors of the Laplacian as $𝐟=\sum_{i}a_{i}{𝐮}_{i}$, where ${𝐮}_{i}$ is the *i*-th column of the matrix 𝐔 and $𝐚={𝐔}^{⊤}𝐟$ is known as the Graph Fourier Transform of **f** [28]. This gives an alternative way to express the quadratic form of Eq (1).

$${𝐟}^{⊤}𝐋𝐟={𝐚}^{⊤}\Lambda_{L}𝐚=\sum_{i=1}^{N}a_{i}^{2}\lambda_{i}^{L}$$ (2)

**Fig 1:**
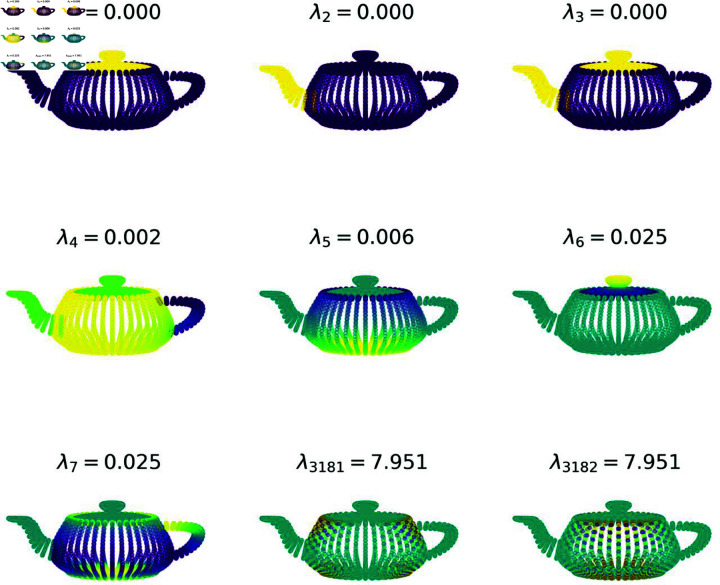
Colour representation of seven low-frequency eigenvectors and two high-frequency eigenvectors of a 3D mesh graph, along with their associated eigenvalue. The mesh is constructed from the classic Utah Teapot 3D object. Here, the main body, lid and spout are all mutually disconnected, giving three zero eigenvalues.

These two facts provide the interpretation of the eigenvectors of the Laplacian as having varying degrees of smoothness, ordered by the size of the associated eigenvalue. The number of zero eigenvalues is the number of disconnected subgraphs. Beyond that, larger eigenvalues, analogous to frequency in classical signal processing, are associated with increasingly ‘rough’ eigenvectors. Fig 1 shows an example of this behaviour for a 3D mesh graph.

### 2.2 Graph filters

The concept of a graph filter can be constructed by extending the analogy of the GFT [29]. A low-pass filter, 𝐇, which operates on a graph signal **f**, attenuates the high-frequency graph Fourier components and can be used to smooth or denoise a signal [30]. It can be constructed by defining a decreasing function η(λ;β), parametrised by $\beta \in {\mathbb{R}}^{+}$, though we will suppress the parameter when using the function unless explicitly required, for which η(0)=1 and $\lim_{\lambda \to \infty }\eta (\lambda )=0$. The filter, 𝐇, is then defined by

$$𝐇=𝐔\eta (\Lambda_{L})\;{𝐔}^{⊤}$$ (3)

where $\eta (\Lambda_{L})$ denotes the application of η(·) element wise to the diagonal. The filtered signal ${𝐟}^{\prime }$ is then calculated as 𝐇𝐟. Note that the operator 𝐇 is necessarily symmetric and semi-positive definite. Numerous possibilities exist for η(λ), some of which are outlined in Table 2.

**Table 2:** Possible graph filter functions.

Filter	η(λ;β)	Parameter
Inverse	$(1+\beta \lambda {)}^{-1}$	$\beta \in {\mathbb{R}}^{+}$
Exponential	exp(−βλ)	$\beta \in {\mathbb{R}}^{+}$
ReLu	max(1−βλ,0)	$\beta \in {\mathbb{R}}^{+}$
Sigmoid	$2(1+\exp(\beta \lambda ){)}^{-1}$	$\beta \in {\mathbb{R}}^{+}$
Cosine	$\cos^{\;\beta }(\lambda \pi /2\lambda_{\text{max}})$	$\beta \in {\mathbb{R}}^{+}$
Cut-off	1,ifλ≤βelse0	$\beta \in {\mathbb{R}}^{+}$

Graph filters constructed in this way bear a close resemblance to graph kernels as described in [18], where instead the authors define an increasing function r(λ). In practical terms, a graph filter can be considered the inverse of a graph kernel as defined in this paper or, more accurately, $\eta (\lambda )=\lim_{c\to 0}1/(c+r(\lambda ))$. “Graph kernel" in this context should not be confused with another concept of the same name, which concerns distance metrics between pairs of graphs [31].

### 2.3 Graph spectral priors

Graph filters can also be used to construct covariance matrices, for example in [14,32]. Consider a Gaussian white noise graph signal ${𝐟}_{\text{w}}$ with precision γ. If a filter 𝐇 is applied to give ${𝐟}_{\text{g}}=𝐇{𝐟}_{\text{w}}$, the resultant signal will be smoother with respect to the graph and will be drawn from a distribution

$${𝐟}_{\text{g}}\sim 𝒩(0,\gamma^{-1}{𝐇}^{2})$$ (4)

In a Bayesian setting, ${𝐇}^{2}$ can act as a covariance matrix which encodes the assumption that signals observed over a graph are likely to be smooth with respect to the underlying topology.

The selection of an appropriate graph filter in this context can be guided by prior knowledge about the expected power spectrum. Adaptive techniques have also been proposed for jointly learning a graph filter such as [15,33] but are not considered in this paper.

## 3. Kernel graph regression

### 3.1 Data and Definitions

This problem runs over a set of *T* discrete “times" 𝒯={1,2,...T}. (These need not be regularly-sampled time intervals but in many practical problems will be). At each time, there exists a fixed graph 𝒢 with *N* nodes as specified earlier by vertex set 𝒩 and related by a static adjacency matrix $𝐀\in {\mathbb{R}}^{N\times N}$.

At a distinct subset of times ${𝒯}^{\prime }\subset 𝒯$ where $|{𝒯}^{\prime }|=T^{\prime }$ we observe supervised learning pairs $\{{𝐱}_{t},{𝐲}_{t}{\}}_{t\in {𝒯}^{\prime }}$. Here, ${𝐱}_{t}\in {\mathbb{R}}^{M}$ is a vector of explanatory variables, and ${𝐲}_{t}\in {\mathbb{R}}^{N^{\prime }}$ is a partially observed graph signal. This signal is measured on a subset of the nodes ${𝒩}^{\prime }\subset 𝒩$ where $|{𝒩}^{\prime }|\;=\;N^{\prime }$ which remains constant over all $t\in {𝒯}^{\prime }$. In a sensor network, this may be the case if only a subset of the sensors are functioning correctly, or we wish to estimate the reading at locations where no physical sensor is placed. The goal is to predict the graph signal at the unobserved nodes for $t\in {𝒯}^{\prime }$ and at all nodes for $t\in 𝒯-{𝒯}^{\prime }$.

It is useful to define two binary selection matrices ${𝐒}_{N}\in [0,1{]}^{N^{\prime }\times N}$ and ${𝐒}_{T}\in [0,1{]}^{T^{\prime }\times T}$ which help in mapping between observed and unobserved node sets. ${𝐒}_{N}$ has $\left(n^{\prime },n\right)$-th entry equal to one for $n^{\prime }=1$ to $n^{\prime }=N^{\prime }$ and $n\in {𝒩}^{\prime }$, with the rest set to zero. Similarly ${𝐒}_{T}$ has entries $\left(t^{\prime },t\right)$ equal to one for $t^{\prime }=1$ to $t^{\prime }=T^{\prime }$ and $t^{\prime }\in {𝒯}^{\prime }$, with the rest set to zero. For simplicity, and without loss of generality, we can assume that

$${𝐒}_{N}=[\begin{array}{cc} {𝐈}_{N^{\prime }} & 0_{N^{\prime }\times (N-N^{\prime })} \end{array}]\in [0,1{]}^{N^{\prime }\times N}\;{𝐒}_{T}=[\begin{array}{cc} {𝐈}_{T^{\prime }} & \;0_{T^{\prime }\times (T-T^{\prime })}\; \end{array}]\in [0,1{]}^{T^{\prime }\times T}\;$$ (5)

which implies that the first $N^{\prime }$ nodes are observed, and the first $T^{\prime }$ times contain the labelled examples. Algebraically, this is always possible under a reordering of nodes/times. However, statistically, depending on the regression assumptions of the model, one should note that such reordering may influence the regression results, depending on what assumptions on the autocorrelation structures and conditional dependence assumptions of the response variables, given the covariates, are made for the observations over time in the graph regression. If the observations are assumed conditionally uncorrelated (or independent if under a Gaussian error assumption) in time, given the covariates, then reordering in this manner will not influence the regression results, in the case that the covariates time series $\left\{{𝐱}_{t}\right\}$ does not display autocorrelation or cross autocorrelation in any coordinates (of course assuming covariates in the regression are also reordered accordingly). If however, the covariates do have a temporal cross correlation then mixing assumptions may need to be considered when deciding about the suitability of reordering, such that reordering takes place only between time intervals with lags that are sufficiently uncorrelated in the design space covariate time series. If additionally, there is deemed to be a conditional autocorrelation in the response time series, even after conditioning on regressors, in the graph regression, then such reordering should be considered carefully as it may influence the results of the regression including the smoothness of signals learnt. In this instance, one may perform a decorrelating transformation on the observation covariates prior to performing the reordering, of course, this would assume knowledge of the conditional autocorrelation matrix of the response in the regression. When this is not known, it leads to a generalised iterative Graph regression that is the extension of Generalised Least Squares (see [34]) into the graph regression setting. Such an example is illustrated in the AR(1) error process in Section 4.3 below. Alternatively, if the assumptions required to perform re-ordering are not easily satisfied for a given regression application, then there is no problem with working with matrices ${𝐒}_{N}$ and ${𝐒}_{T}$ without any reordering, it is just less convenient algebraically.

### 3.2 KGR via signal reconstruction

We will now derive a Kernel Graph Regression model from the perspective of graph signal smoothing and reconstruction. Let us assume that there exists an underlying noiseless graph signal ${𝐟}_{t}\in {\mathbb{R}}^{N}$ at each time that we wish to estimate. The observation ${𝐲}_{t}$ is modelled as a partial noisy observation of ${𝐟}_{t}$. This statement is summarised by the following model.

$$𝐘={𝐒}_{N}𝐅{𝐒}_{T}^{⊤}+𝐄.$$ (6)

Here $𝐘\in {\mathbb{R}}^{N^{\prime }\times T^{\prime }}$ is a horizontal stacking of each partial graph observation such that the *t*-th column is the partial graph signal observed at time *t*, and the *n*-th row is the time series observed at node *n*. $𝐅\in {\mathbb{R}}^{N\times T}$ has a similar structure, but represents the true underlying function at the full, length-*N*, node-set at all times. $𝐄\in {\mathbb{R}}^{N^{\prime }\times T^{\prime }}$ is an error matrix of standard normal i.i.d. noise. The probability distribution of 𝐘 can therefore be expressed as

$$\text{vec}(𝐘)\;|\;𝐅\sim 𝒩(\text{vec}({𝐒}_{N}𝐅{𝐒}_{T}^{⊤}),\;{𝐈}_{T^{\prime }}⊗{𝐈}_{N^{\prime }})$$ (7)

where vec(·) has the usual meaning of a vertical stacking of matrix columns, and ⊗ is the Kronecker product. In order to estimate the latent signal 𝐅, we must specify a prior distribution indicating our belief at the outset about the likelihood of different signals. This provides the necessary Tikhonov regularization term and avoids under-specification. In this case, an appropriate prior for 𝐅, which has also been used in [14], is the following

$$\text{vec}(𝐅)\;|\;𝐗\sim 𝒩(0,\;\gamma^{-1}𝐊⊗{𝐇}^{2})$$ (8)

where matrix 𝐇 is a filtered graph Laplacian matrix, as defined in Equation (3), for a user specified graph filter η(·), such as one of those specified in Table 2 and γ is a precision parameter which controls the regularisation strength.

Here, $𝐊\in {\mathbb{R}}^{T\times T}$ is a kernel (or Gram) matrix defined by the relation ${𝐊}_{ij}=\kappa ({𝐱}_{i},{𝐱}_{j};\sigma )$, where κ(·,·;σ) is a Mercer kernel (see [35]) with parameter(s) σ. In a kernel matrix 𝐊, the entries represent the inner product between pairs of (potentially infinite-dimensional) basis function representations of the features. That is, ${𝐊}_{ij}=\langle \phi ({𝐱}_{i}),\phi ({𝐱}_{j})\rangle$. An expample of a typical kernel used in machine learning is the Gaussian kernel:

$$\kappa ({𝐱}_{i},{𝐱}_{j};\sigma )=\exp(-\frac{|{𝐱}_{i}-{𝐱}_{j}{|}^{2}}{2\sigma^{2}})$$ (9)

A natural question to ask is why we use the prior given in Eq (8), with a covariance matrix given by the Kronecker product between a kernel matrix and a squared graph filter. Intuitively, it makes sense that the correlation between two node-times $\left(n_{1},t_{1}\right)$ and $\left(n_{2},t_{2}\right)$ is expected to be high when the nodes are closely connected and the explanetory variables are similar, i.e. $|{𝐱}_{t_{1}}$ − ${𝐱}_{t_{2}}|$ is small. Eq (8) directly maps onto this intuition, since the prior covariance between two signal elements ${𝐅}_{n_{1},t_{1}}$ and ${𝐅}_{n_{2},t_{2}}$ is given by


$$\text{Cov}[{𝐅}_{n_{1},t_{1}},{𝐅}_{n_{2},t_{2}}]=\gamma^{-1}\kappa ({𝐱}_{t_{1}},{𝐱}_{t_{2}})({𝐇}^{2}{)}_{n_{1},n_{2}}$$

In the supplementary materials, we provide a more formal justification for why this prior is appropriate. This is derived by considering the “weight-space" view of multivariate Gaussian processes [36]. This derivation begins with a multivariate linear regression model with a spherical prior over the regression coefficients. Then, the so-called ‘kernel trick’ in used to translate this into a non-parametric model with a kernel matrix 𝐊. The only substantial modification to this original derivation that we make is to place a graph spectral prior over the regression coefficients, rather than the usual spherical prior. This encourages predictions which are smooth with respect to the graph topology.

Applying Bayes’ rule to Eqs (7) and (8) results in the following Maximum a Posteriori (MAP) optimisation problem for 𝐅.


$${𝐅}^{⋆}=\underset{𝐅}{\text{argmin}}\;[-\ln\pi (𝐅|𝐘,𝐗)]=\underset{𝐅}{\text{argmin}}\;[\xi (𝐅)]$$

where we use the notation π(·) to represent a probability density function. Using the definition of the density function for a matrix normal distribution, ξ(𝐅) can be written as

$$\xi (𝐅)=\text{tr}(\left(𝐘-{𝐒}_{N}𝐅{𝐒}_{T}^{⊤}{)}^{⊤}(𝐘-{𝐒}_{N}𝐅{𝐒}_{T}^{⊤}\right))+\gamma \;\text{tr}({𝐊}^{-1}{𝐅}^{⊤}{𝐇}^{-2}𝐅)$$ (10)

Taking the derivative of this expression with respect to 𝐅 and setting the result to zero yields a solution for ${𝐅}^{⋆}$. We refer to this as the KGR solution, but can also equivalently be considered the mean of a GPoG solution.

$$\text{vec}({𝐅}^{⋆})=({𝐒}_{T}^{⊤}{𝐒}_{T}⊗{𝐒}_{N}^{⊤}{𝐒}_{N}+\gamma {𝐊}^{-1}⊗{𝐇}^{-2}{)}^{-1}({𝐒}_{T}^{⊤}⊗{𝐒}_{N}^{⊤})\text{vec}(𝐘)$$ (11)

Applying a well-known matrix identity ([37], Eq 162) allows the dimension of the inverse to be reduced from NT×NT to $N^{\prime }T^{\prime }\times N^{\prime }T^{\prime }$.

$$\text{vec}({𝐅}^{⋆})=(𝐊{𝐒}_{T}^{⊤}⊗{𝐇}^{2}{𝐒}_{N}^{⊤})(\gamma {𝐈}_{T^{\prime }}⊗{𝐈}_{N^{\prime }}+\bar{𝐊}⊗\bar{{𝐇}^{2}}{)}^{-1}\text{vec}(𝐘)$$ (12)

where $\bar{𝐊}={𝐒}_{T}𝐊{𝐒}_{T}^{⊤}$ and $\bar{{𝐇}^{2}}={𝐒}_{N}{𝐇}^{2}{𝐒}_{N}^{⊤}$. However, in this form, computation is impractical for large problems (*T* or *N* or both large) due to the high complexity involved in inverting the Kronecker-structured matrix given by the form $\left(\gamma {𝐈}_{T^{\prime }}⊗{𝐈}_{N^{\prime }}+\bar{𝐊}⊗\bar{{𝐇}^{2}}\right)$. Thankfully this can be overcome by performing eigendecomposition on $\bar{𝐊}$ and $\bar{{𝐇}^{2}}$ separately and leveraging properties of the Kronecker product. For a detailed derivation, consult the supplementary materials. Using the notation ∘ to represent the Hadamard product, the result is

$${𝐅}^{⋆}={𝐇}^{2}{𝐒}_{N}^{⊤}\bar{𝐔}(𝐉\circ ({\bar{𝐔}}^{⊤}𝐘\bar{𝐕})){\bar{𝐕}}^{⊤}{𝐒}_{T}𝐊$$ (13)

where

$$\bar{𝐊}=\bar{𝐕}{\bar{\Lambda }}_{K}{\bar{𝐕}}^{⊤},\;\text{and}\;\bar{{𝐇}^{2}}=\bar{𝐔}{\bar{\Lambda }}_{H}{\bar{𝐔}}^{⊤}$$ (14)

and $𝐉\in {\mathbb{R}}^{N^{\prime }\times T^{\prime }}$ has the elements given by

$${𝐉}_{ij}=\frac{1}{{\bar{\lambda }}_{j}^{K}{\bar{\lambda }}_{i}^{H}+\gamma }$$ (15)

A key benefit of the solution in this form is that the necessary dense eigendecompositions, which are typically the most computationally taxing step, are performed on $\bar{𝐊}$ and $\bar{{𝐇}^{2}}$, of size $T^{\prime }\times T^{\prime }$ and $N^{\prime }\times N^{\prime }$ respectively, whereas a naive implementation would require this to be T×T and N×N. This can be a significant speed-up for large problems, especially when a meaningful portion of the data is unlabelled. (Decomposition of $𝐋\in {\mathbb{R}}^{N\times N}$ is also required, but this is typically sparse so can often be accelerated using sparsity-specific linear algebra tools, see [38] and references therein).

A full outline of all the steps for graph kernel regression is highlighted in algorithm 1.



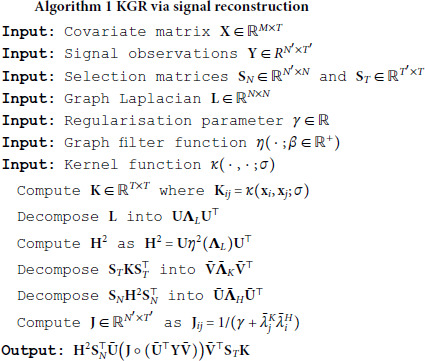



### 3.3 KGR and Cartesian product graphs

In this short section, we highlight the connection between Kernel Graph Regression and Graph Signal Reconstruction (GSR). In particular, the mathematical formalism of KGR described so far in this paper bears a strong resemblance to Bayesian signal reconstruction as applied to a Cartesian product graph. The reconstruction of signals defined over Cartesian product graphs is an area that has received increasing attention in recent years [39,40], particularly in the context of Time-Vertex (T-V) problems [41]. By adapting the KGR algorithm slightly, we effectively get a signal reconstruction algorithm with little effort. This also highlights the connection between KGR and GSR which can provide insight into both areas.

**Fig 2:**
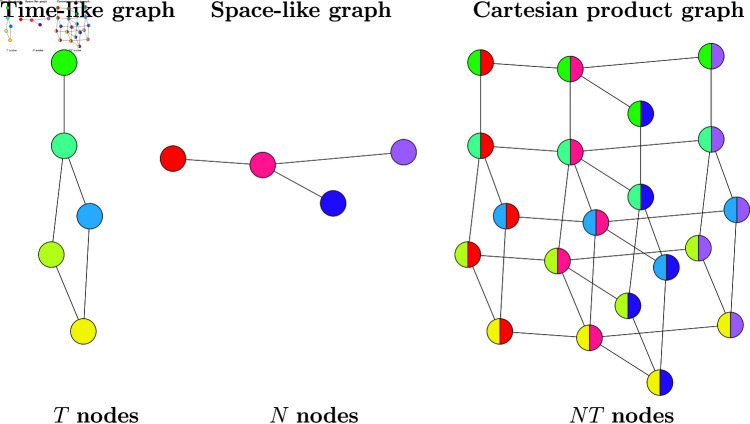
A graphical depiction of a small Cartesian product graph.

A Cartesian product graph is described by two adjacency matrices ${𝐀}_{T}\in {\mathbb{R}}^{T\times T}$ and ${𝐀}_{N}\;\in \;{\mathbb{R}}^{N\times N}$ which, in the following, we refer to as the ‘time-like’ graph and the ‘space-like’ graph respectively. Fig 2 gives a graphical depiction of a small Cartesian product graph. The resultant graph has *NT* nodes, with an adjacency matrix $𝐀\in {\mathbb{R}}^{NT\times NT}$, given by the Kronecker sum of the two individual adjacency matrices, specified as

$$𝐀={𝐀}_{T}⊕{𝐀}_{N}={𝐀}_{T}⊗{𝐈}_{N}+{𝐈}_{T}⊕{𝐀}_{N}.$$ (16)

Similarly, the Laplacian matrix of the Cartesian product, 𝐋, is given by the Kronecker sum of the individual Laplacian matrices, that is

$$𝐋={𝐋}_{T}⊕{𝐋}_{N}$$ (17)

The Laplacian can be eigendecomposed as follows.

$$𝐋=({𝐔}_{T}⊗{𝐔}_{N})(\Lambda_{T}⊕\Lambda_{N})({𝐔}_{T}^{⊤}⊗{𝐔}_{N}^{⊤})$$ (18)

where ${𝐋}_{T}={𝐔}_{T}\Lambda_{T}{𝐔}_{T}^{⊤}$ and ${𝐋}_{N}={𝐔}_{N}\Lambda_{N}{𝐔}_{N}^{⊤}$. Therefore, a general graph filter 𝐇 associated with the product graph can be understood as applying a decreasing function η(·) to the diagonal eigenvalue matrix $\Lambda_{T}⊕\Lambda_{N}$.

$$𝐇=({𝐔}_{T}⊗{𝐔}_{N})\eta (\Lambda_{T}⊕\Lambda_{N})({𝐔}_{T}^{⊤}⊗{𝐔}_{N}^{⊤})$$ (19)

For a certain class of graph filter functions, for example, the exponential filter, it is the case that

$$\eta (\Lambda_{T}⊕\Lambda_{N})=\eta (\Lambda_{T})⊗\eta (\Lambda_{N})$$ (20)

We refer to graph filters of this type as separable. If this condition holds, it implies that the total graph filter can also be expressed as a Kronecker product.

$$𝐇={𝐇}_{T}⊗{𝐇}_{N}$$ (21)

where ${𝐇}_{T}={𝐔}_{T}\eta (\Lambda_{T}){𝐔}_{T}^{⊤}$ and ${𝐇}_{T}={𝐔}_{N}\eta (\Lambda_{N}){𝐔}_{N}^{⊤}$. Now consider a graph signal reconstruction problem, where the task is to estimate a smooth underlying signal $𝐅\in {\mathbb{R}}^{N\times T}$, given a signal $𝐘\in {\mathbb{R}}^{N^{\prime }\times T^{\prime }}$ observed over a constant subset of the space-like and time-like nodes. Once again, we assume that 𝐘 is a noisy partial observation of 𝐅 given by Eq (6). The only key difference from the KGR formulation is that the prior distribution for 𝐅 is no longer given by Eq (8), but instead is given by

$$\text{vec}(𝐅)\sim 𝒩(0,\gamma^{-1}{𝐇}_{T}⊗{𝐇}_{N})$$ (22)

Note that the only material difference is that the kernel matrix 𝐊 has been replaced by another filter matrix ${𝐇}_{T}$. Following the Bayesian logic of the previous section immediately leads to a graph signal reconstruction method, given explicitly in algorithm 2.



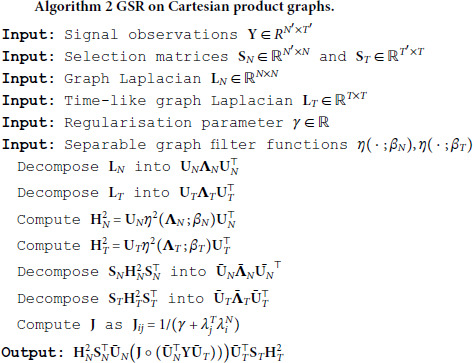



## 4 GLS kernel graph regression

In many real-world applications of regression modelling, the assumption that the error terms of Eq (6) are independent and identically distributed is unlikely to hold. In this section, we consider the situation where the differences between the observed and underlying signals are instead correlated according to a matrix normal distribution. Under this model, the elements of 𝐘 are distributed as

$$\text{vec}(𝐘)|𝐅\sim 𝒩(\text{vec}({𝐒}_{N}𝐅{𝐒}_{T}^{⊤}),\;\Sigma_{T}⊗\Sigma_{N})$$ (23)

where $\Sigma_{T}\in {\mathbb{R}}^{T^{\prime }\times T^{\prime }}$ and $\Sigma_{N}\in {\mathbb{R}}^{N^{\prime }\times N^{\prime }}$ respectively represent the time correlation and vertex covariance matrices. We choose to parameterise the covariance matrix in this way for several reasons. Firstly, some method for reducing the dimensionality of the problem is certainly necessary for the estimation of the matrix. Since the available data to make the estimate is a single $\left(N^{\prime }\times T^{\prime }\right)$ observation 𝐘, estimating a full $\left(N^{\prime }T^{\prime }\times N^{\prime }T^{\prime }\right)$ matrix would not be possible without other regularisation assumptions. Secondly, expressing the covariance matrix as a Kronecker product of two smaller matrices enables tractable estimators. This is primarily due to the property of Kronecker products that $(𝐀⊗𝐁{)}^{-1}={𝐀}^{-1}⊗{𝐁}^{-1}$. In addition, holding a dense $\left(N^{\prime }T^{\prime }\times N^{\prime }T^{\prime }\right)$ matrix in memory may be out of the question. Thirdly, the Kronecker product assumption has an intuitive interpretation in terms of independent factor-specific contributions to the overall covariance. Put simply, the covariance between node-times $\left(n_{1},t_{1}\right)$ and $\left(n_{2},t_{2}\right)$ will be $(\Sigma_{T}{)}_{\left(t_{1},t_{2}\right)}$ × $(\Sigma_{N}{)}_{\left(n_{1},n_{2}\right)}$. If there is no correlation between two times *t*_1_ and *t*_2_, then there should be no correlation between node-times $\left(n_{1},t_{1}\right)$ and $\left(n_{2},t_{2}\right)$, no matter what the value of $(\Sigma_{N}{)}_{\left(n_{1},n_{2}\right)}$. Similarly, if the two nodes are totally uncorrelated, then there should be no correlation between node-times $\left(n_{1},t_{1}\right)$ and $\left(n_{2},t_{2}\right)$, no matter what the value of $(\Sigma_{T}{)}_{\left(t_{1},t_{2}\right)}$. The actual value, $(\Sigma_{T}{)}_{\left(t_{1},t_{2}\right)}$ × $(\Sigma_{N}{)}_{\left(n_{1},n_{2}\right)}$ can be seen as counting the contribution from both the time correlation and the node-level correlation multiplicatively. Finally, this model has also been used in many applications relevant to network problems such as geospatial, econometric, and EEG models [42–44]. For other recent approaches, see [45,46].

### 4.1 KGR with Gauss–Markov estimator

To begin, we derive a Gauss–Markov estimator for 𝐅, that is, the Best Linear Unbiased Estimator (BLUE) assuming that the covariance matrix $\Sigma_{T}$ ⊗ $\Sigma_{N}$ is known. (This restriction is relaxed in the subsequent section). In this case, the log-likelihood of making an observation 𝐘 is altered such that the cost function of Eq (10) becomes

$$\xi (𝐅)=\text{tr}((𝐘-{𝐒}_{N}𝐅{𝐒}_{T}^{⊤}{)}^{⊤}\Sigma_{N}^{-1}(𝐘-{𝐒}_{N}𝐅{𝐒}_{T}^{⊤})\;\Sigma_{T}^{-1})+\gamma \;\text{tr}({𝐊}^{-1}{𝐅}^{⊤}{𝐇}^{-2}𝐅)$$ (24)

The BLUE estimator for a given $\Sigma_{N}$ and $\Sigma_{T}$ is defined to be the value of 𝐅 which minimises this expression and can be found by differentiating it with respect to 𝐅 and setting the result equal to zero. By again following the same steps as in section 3, this procedure results in

$$\text{vec}({𝐅}^{⋆})=({𝐒}_{T}^{⊤}\Sigma_{T}^{-1}{𝐒}_{T}⊗{𝐒}_{N}^{⊤}\Sigma_{N}^{-1}{𝐒}_{N}+\gamma {𝐊}^{-1}⊗{𝐇}^{-2}{)}^{-1}\;\times ({𝐒}_{T}^{⊤}\Sigma_{T}^{-1}⊗{𝐒}_{N}^{⊤}\Sigma_{N}^{-1})\;\text{vec}(𝐘)$$ (25)

Once again, in this form, the solution remains prohibitively expensive to compute for large $N^{\prime }$ and $T^{\prime }$. However, it can be expressed alternatively with $O(N^{\prime 3}+T^{\prime 3})$ complexity by making the following definitions. First eigendecompose $\Sigma_{N}$ and $\Sigma_{T}$.


$$\Sigma_{T}=\Psi \Lambda_{\Sigma_{T}}\Psi^{⊤},\;\Sigma_{N}=\Phi \Lambda_{\Sigma_{N}}\Phi^{⊤}$$

Then perform eigendecomposition on the following matrices:


$$\Lambda_{\Sigma_{T}}^{-1/2}\Psi^{⊤}{𝐒}_{T}𝐊{𝐒}_{T}^{⊤}\Psi \Lambda_{\Sigma_{T}}^{-1/2}=\bar{𝐕}{\bar{\Lambda }}_{K}{\bar{𝐕}}^{⊤}\;\Lambda_{\Sigma_{N}}^{-1/2}\Phi^{⊤}{𝐒}_{N}{𝐇}^{2}{𝐒}_{N}^{⊤}\Phi \Lambda_{\Sigma_{N}}^{-1/2}=\bar{𝐔}{\bar{\Lambda }}_{H}{\bar{𝐔}}^{⊤}$$

Note that both these matrices are guaranteed to be symmetric with positive, real eigenvalues [47]. Finally, make the following definitions:


$${𝐉}_{ij}=\frac{1}{\gamma +{\bar{\lambda }}_{j}^{K}{\bar{\lambda }}_{i}^{H}}\;𝐁={𝐇}^{2}{𝐒}_{N}^{⊤}\Phi \Lambda_{\Sigma_{N}}^{-1/2}\bar{𝐔}\;𝐂=𝐊{𝐒}_{T}^{⊤}\Psi \Lambda_{\Sigma_{T}}^{-1/2}\bar{𝐕}\;\bar{𝐘}={\bar{𝐔}}^{⊤}\Lambda_{\Sigma_{N}}^{-1/2}\Phi^{⊤}𝐘\Psi \Lambda_{\Sigma_{T}}^{-1/2}\bar{𝐕}$$

The GLS estimator for ${𝐅}^{⋆}$ is given by

$${𝐅}^{⋆}=𝐁\;(𝐉\circ \bar{𝐘})\;{𝐂}^{⊤}$$ (26)

While a simpler *O*(*N*^3^  +  *T*^3^) solution is possible, the above method ensures that the eigendecompositions are performed on reduced size matrices, effectively scaling as $O(N^{\prime 3}$  +  $T^{\prime 3})$. For a detailed derivation with intermediate steps, we refer the reader to the supplementary materials.



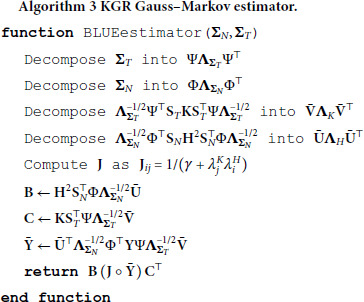



### 4.2 Estimating $\Sigma_{N}$SigN and $\Sigma_{T}$SigT given 𝐅F

In this section, we construct estimators for the covariance matrices $\Sigma_{N}$ and $\Sigma_{T}$, given that the latent signal 𝐅, and therefore the prediction error $𝐄=𝐘-{𝐒}_{N}𝐅{𝐒}_{T}^{⊤}$, is known. It follows from Eq (23), that 𝐄 has a matrix normal distribution of the following form.

$$\text{vec}(𝐄)\sim 𝒩(0,\;\Sigma_{T}⊗\Sigma_{N})$$ (27)

Past literature on parameter estimation in matrix-normal models has mostly focused on the task of estimating the covariance given multiple realisations of the error matrix 𝐄 [48–51]. In order to estimate both $\Sigma_{N}$ and $\Sigma_{T}$ in full, the number of observations of 𝐄 must be greater than [max$\left(N^{\prime }/T^{\prime },T^{\prime }/N^{\prime }\right)$  +  1] [52]. However, in the present case, only a single observation comprising $N^{\prime }T^{\prime }$ values is available. It is therefore necessary to assume a simplified, parametrised structure for at least one of these matrices to reduce the number of degrees of freedom.

For this reason, we assume that the matrix $\Sigma_{T}(\theta )$ describes the autocorrelation between different time measurements and is a function of a parameter or set of parameters θ. The elements of $\Sigma_{T}(\theta )$ are constrained to be dimensionless Pearson correlation coefficients, with 1 along the diagonal. Conversely, $\Sigma_{N}$ is assumed to be an arbitrary covariance matrix that can take on any value satisfying semi-positive definiteness. By explicitly parametrising one as a correlation matrix, parameterised by a function of a parameter vector θ in much lower dimension than *T*, while letting the other act as a fully parameterised covariance matrix, the problem of the solution being under-determined (since $a\Sigma_{T}⊗\frac{1}{a}\Sigma_{N}$ gives the same matrix for any *a*) is avoided.

The likelihood of observing an error matrix 𝐄, given covariance matrices $\Sigma_{N}$ and $\Sigma_{T}(\theta )$, is

$$\pi (𝐄|\theta ,\Sigma_{N})=2\pi^{\frac{NT}{2}}(\;|\Sigma_{T}(\theta )⊗\Sigma_{N}|\;{)}^{-1/2}\exp(-\frac{1}{2}\text{tr}({𝐄}^{⊤}\Sigma_{N}^{-1}𝐄\;\Sigma_{T}^{-1}(\theta )))$$ (28)

By specifying two independent prior distributions π(θ) and $\pi (\Sigma_{N})$, the posterior density over θ and $\Sigma_{N}$ can be expressed via Bayes’ rule as


$$\pi (\theta ,\Sigma_{N}|𝐄)=\frac{\pi (𝐄|\theta ,\Sigma_{N})\;\pi (\theta )\;\pi (\Sigma_{N})}{\pi (𝐄)}$$

Depending on the size of the problem, this could be used along with a Monte Carlo algorithm to sample from the whole posterior or, more likely, be used to calculate single MAP estimates for θ and $\Sigma_{N}$. In either case, the negative log posterior, up to a scaling and additive constant, is equal to

$$\xi (\theta ,\Sigma_{N})=N^{\prime }\ln|\Sigma_{T}(\theta )|+T^{\prime }\ln|\Sigma_{N}|+\text{tr}({𝐄}^{⊤}\Sigma_{N}^{-1}𝐄\;\Sigma_{T}^{-1}(\theta ))\;-2\ln\pi (\theta )-2\ln\pi (\Sigma_{N})$$ (29)

A MAP estimate can be found by minimising this quantity jointly with respect to the parameters. There are several possible strategies for solving this, such as simple gradient descent. However, since an analytical solution for θ is often possible given $\Sigma_{N}$ and vice versa, an efficient algorithm using temporary approximate solutions can often be found. This so-called ‘flip-flop’ strategy is widely used in the literature on Kronecker-product covariance estimation [52]. Crucially, the number of iterations required for convergence does not depend greatly on the size of the system, meaning $O(N^{3}+T^{3})$ complexity is retained.

### 4.3 Example: AR(1) process

The previous section stated the general formulation of the problem without any particular parametrisation of the matrix $\Sigma_{T}(\theta )$. In this section, we give a concrete example by considering the widely used AR(1) model, which assumes simple, serial correlation between prediction errors. Denoting the *t*-th column of 𝐄 as ${𝐄}_{t}$, the general AR(1) model assumes that

$${𝐄}_{t}=\Theta \;{𝐄}_{t-1}+\varepsilon_{t},\;\varepsilon_{t}\sim 𝒩(0,\Sigma_{N}),$$ (30)

where $\Theta \in {\mathbb{R}}^{N\times N}$ is a matrix of autoregression coefficients [53]. In the following, we assume a simplified version, where the *n*-th element of ${𝐄}_{t}$ is assumed only to be serially correlated with the *n*-th element of ${𝐄}_{t-1}$, in a way that is both stationary and uniform across all *n*. In effect, this requires that $\Theta =\theta {𝐈}_{N}$. This is, in essence, the multivariate extension of the AR(1) model considered in the seminal paper by Cochrane and Orcutt [54]. In the limit as T→∞, the correlation matrix $\Sigma_{T}$, and its inverse $\Sigma_{T}^{-1}$, have a well-known form, which can be truncated approximately for large enough *T* to the form given by


$$\Sigma_{T}(\theta )=[\begin{array}{ccccc} 1 & \theta & \theta^{2} & \cdots & \theta^{T^{\prime }-1}\\ \theta & 1 & \theta & \cdots & \theta^{T^{\prime }-2}\\ \vdots & \vdots & \vdots & \ddots & \vdots \\ \theta^{T^{\prime }-1} & \theta^{T^{\prime }-2} & \theta^{T^{\prime }-3} & \cdots & 1 \end{array}],$$

$$\Sigma_{T}^{-1}(\theta )=\frac{1}{1-\theta^{2}}[\begin{array}{cccccc} 1 & -\theta & & & 0\\ -\theta & 1+\theta^{2} & -\theta & & & \\ & \ddots & \ddots & \ddots & & \\ & & -\theta & 1+\theta^{2} & -\theta \\ 0 & & & -\theta & 1 \end{array}]$$

$$=\frac{1}{1-\theta^{2}}({𝐈}_{T^{\prime }}-\theta {𝐁}_{1}+\theta^{2}{𝐁}_{2}),$$ (31)

where ${𝐁}_{1}$ is a matrix with 0 on the diagonal and with 1 on the first upper and lower diagonal, and ${𝐁}_{2}$ is the identity matrix with zero on the first and last diagonal entries [55]. Note also that the determinant of $\Sigma_{T}$ is given by $(1-\theta^{2}{)}^{T^{\prime }-1}$ [56]. To be a valid stationary process, the parameter θ must lie on the interval (–1,1). An appropriate prior distribution for θ could therefore be

$$\pi (\theta |\alpha )\propto \exp(-\frac{N^{\prime }T^{\prime }\alpha /2}{1-\theta^{2}}),\;\text{for}\;\theta \in (-1,1)$$ (32)

for some scalar parameter α. Depending on the value of α, this prior is roughly uniform across the majority of the interval, whilst rapidly decreasing in likelihood towards –1 and 1. This effectively encodes a belief that the time series is stationary across all nodes, with α controlling the strength of that stationarity assumption. The resultant θ-dependant part of the cost function of Eq (29) therefore becomes

$$N^{\prime }(T^{\prime }-1)\ln(1-\theta^{2})+\text{tr}({𝐄}^{⊤}\Sigma_{N}^{-1}𝐄\;\Sigma_{T}^{-1}(\theta ))+\frac{N^{\prime }T^{\prime }\alpha }{1-\theta^{2}}$$ (33)

Whilst this is not a quadratic function of θ, the unique maximum likelihood estimator $\hat{\theta }$ can be found by differentiating this expression and setting the result to zero. This results in a MAP estimator which is the real root of a cubic polynomial.

$$\hat{\theta }(\Sigma_{N})=\text{real root}[\;\frac{b}{2}+(N^{\prime }(T^{\prime }-1)-a-c-N^{\prime }T^{\prime }\alpha )\;\theta \;+\frac{b}{2}\theta^{2}-N^{\prime }(T^{\prime }-1)\;\theta^{3}\;],$$ (34)

where

$$a=\text{tr}({𝐄}^{⊤}\Sigma_{N}^{-1}𝐄),\;b=\text{tr}({𝐄}^{⊤}\Sigma_{N}^{-1}𝐄{𝐁}_{1}),\;\text{and}\;c=\text{tr}({𝐄}^{⊤}\Sigma_{N}^{-1}𝐄{𝐁}_{2}).$$ (35)

A derivation of this expression can be found in the supplementary materials. Note that this estimator is a function of $\Sigma_{N}$.

In terms of the cross-covariance matrix $\Sigma_{N}$, we choose to implement a modified form of the estimator proposed in [57] and further developed in [58]. In essence, these papers considered a weighted combination of the high-variance and often ill-posed maximum likelihood estimate, $\Sigma_{\text{ML}}=\frac{1}{T^{\prime }}𝐄{𝐄}^{⊤}$, and the heavily-biased but well-conditioned estimate, $\frac{1}{N^{\prime }}\text{tr}(\Sigma_{\text{ML}}){𝐈}_{N^{\prime }}$. The only modification presented here is the introduction of $\Sigma_{T}^{-1}$ into the ML estimate, that is, $\Sigma_{\text{ML}}=\frac{1}{T^{\prime }}𝐄\Sigma_{T}^{-1}(\theta ){𝐄}^{⊤}$, which is necessary to account for the effect of autocorrelation.

$${\hat{\Sigma }}_{N}(\theta )=(1-\rho )\Sigma_{\text{ML}}+\frac{\rho }{N^{\prime }}\text{tr}(\Sigma_{\text{ML}}){𝐈}_{N^{\prime }}$$ (36)

[57] and [58] also investigate the optimal setting of the shrinkage coefficient ρ, which is a parameter between 0 and 1. Here, we choose to implement the Rao-Blackwell Ledoit-Wolf (RBLW) estimator described in [58]. This is given by


$${\hat{\rho }}_{\text{RBLW}}=\text{min}[\frac{\frac{T^{\prime }-2}{T^{\prime }}\text{tr}(\Sigma_{\text{ML}}^{2})+{\text{tr}}^{2}(\Sigma_{\text{ML}})}{\left(T^{\prime }+2)(\text{tr}(\Sigma_{\text{ML}}^{2})-\frac{1}{N^{\prime }}{\text{tr}}^{2}(\Sigma_{\text{ML}})\right)},\;1]$$

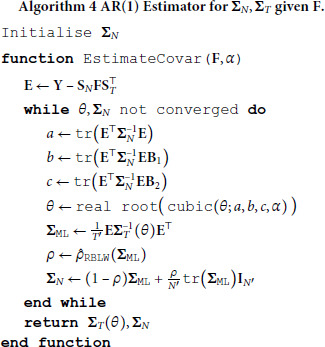



[Eq (34)]

### 4.4 GLS kernel graph regression

To complete the GLS kernel regression algorithm, estimation of both 𝐅 and $\Sigma_{T}$ ⊗ $\Sigma_{N}$ must be performed simultaneously. Since both rely on each other to be estimated, it is necessary to implement an alternating iterative algorithm, where first 𝐅 is estimated for some reasonable initial guess of $\Sigma_{N}$ and $\Sigma_{T}$, and then $\Sigma_{N}$ and $\Sigma_{T}$ are estimated for this value of 𝐅. This process then continues until some convergence criterion is met. These steps are outlined in algorithm 5.

Estimation of the hyperparameters of the kernel is performed using a grid search procedure as discussed further in Section 7.1 below. Additional details of this hyperparameter estimation approach are available in the github repository for the code and detailed worked examples on synthetic and real data, including those contained in this work and additional real data examples, see https://github.com/nickelnine37 and further discussions in [38]. In addition, in the following Section 4.5 explores the model sensitivity to various hyperparameters.

### 4.5 Investigating model sensitivity to hyperparameter selection

In this subsection, we study the effect that the various hyperparameters have on the properties of the GLS-KGR algorithm, using synthetic data. The aim is to provide some intuition for how these variables should be set or learned on real data. The key variables of interest are *γ*, which acts as a global regularisation parameter, *β*, which dictates how smooth the graph signals are expected to be, *α*, which controls the strength of the stationarity assumption, and *σ*, which affects the local variance scale of the Gaussian kernel. We are interested in two key effects that these hyperparameters have on the algorithm, namely, their effect on the prediction accuracy, and their effect on the convergence rate.



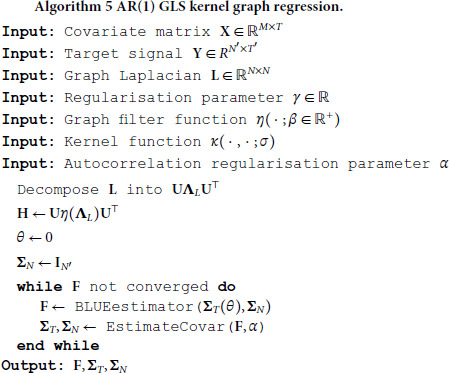



[algorithm 3]

[algorithm 4]

Each experiment was set up in the following way. First, a smooth underlying graph signal 𝐅 was generated by applying a chain-graph diffusion filter 𝐇 to a matrix of Gaussian noise. 20% of the times and nodes were chosen uniformly at random to be hidden, giving two selection matrices ${𝐒}_{N}$ and ${𝐒}_{T}$. A model error matrix 𝐄 was then generated from a matrix normal distribution with known covariance matrices $\Sigma_{N}$ and $\Sigma_{T}$, and added to ${𝐒}_{N}𝐅{𝐒}_{T}^{⊤}$ to create the observed signal 𝐘. Finally, a matrix of Gaussian noise with 12 columns 𝐗 was created to act as the explanatory variables. This model was run under a variety of hyperparameter conditions, with the number of steps required for convergence, and the Root Mean Squared Error (RMSE) on both the seen and unseen node-times recorded on each run.

First, we analyse the convergence properties. The experimental variables γ, β, and σ were found to have a negligible effect on the convergence rate of the GLS Kernel Graph Regression algorithm. That is, when $\Sigma_{N}$, $\Sigma_{T}$ and α remained fixed, varying any of the aforementioned experimental parameters did not affect the number of iterations required to reach a minimum level of precision across the model outputs $\hat{𝐅},{\hat{\Sigma }}_{N}$ and ${\hat{\Sigma }}_{T}$.

On the other hand, we found that $\Sigma_{N}$, $\Sigma_{T}$ and α all had a significant effect on convergence. In order to analyse this systematically, we fixed *N* = 100, *T* = 120, γ=1, β=1 and σ=4. We then varied $\Sigma_{T}(\theta )$ from θ=−0.8 to θ=0.8 in 9 increments of 0.2, and varied α from 10^−3^ to 10^1^ in 50 logarithmic-spaced increments. For each unique θ,α pair we generated 50 unique sets of input data ${𝐒}_{N}$, ${𝐒}_{T}$, 𝐗 and 𝐘 randomly as specified at the start of this section, and ran the GLS KGR model to give 9×50×50=22500 unique trials. For each trial, we counted the total number of iterations required for convergence, including both the inner loop detailed in algorithm 4 and the outer loop of algorithm 5. This experiment was performed twice: In experiment (a) we set $\Sigma_{N}=𝐈$, and in experiment (b) we set ${\Sigma_{N}}_{ij}=\exp((i-j{)}^{2})+0.05\delta_{ij}$, where $\delta_{ij}$ is the Kronecker-delta symbol such that $\delta_{ij}=1$ iff *i* = *j* and $\delta_{ij}=0$ otherwise.

**Fig 3:**
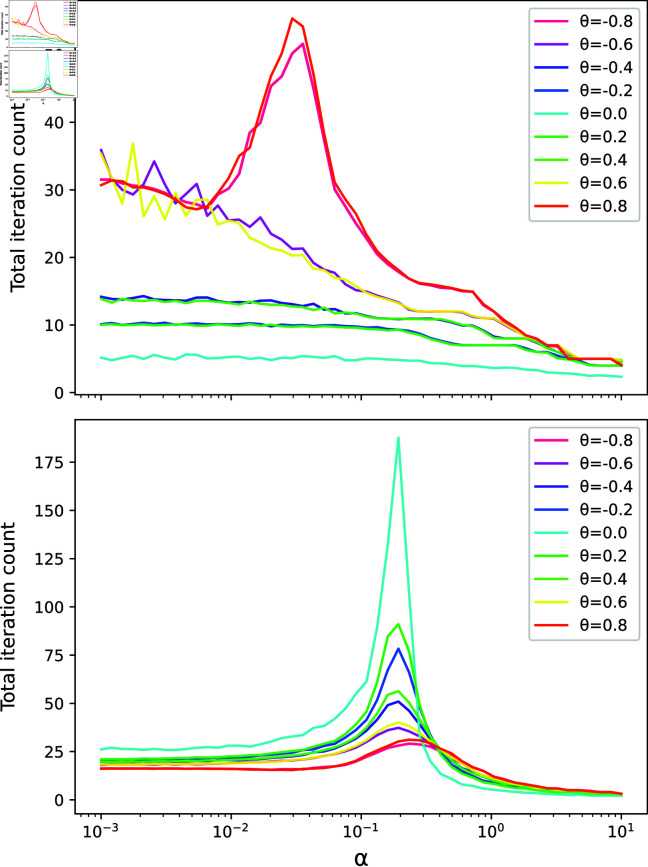
The mean number of iterations required for convergence on synthetic data is shown over a range of values for θ and α. The upper plot shows the results of experiment (a), when $\Sigma_{N}=𝐈$, and the lower plot shows the results for experiment (b), when ${\Sigma_{N}}_{ij}=\exp((i-j{)}^{2})+0.05\delta_{ij}$.

The mean number of iterations required for convergence for each θ,α pair is shown in Fig 3.

In general, we can see that $\Sigma_{N}$, *θ* and *α* have a complex interaction with the number of iterations. In both experiments (a) and (b) we see that convergence is roughly symmetric for ±θ, with this symmetry slightly broken in experiment (b). In both cases, a spike in the number of iterations is observed for a certain value of *α*, although this is not observed in the experimental *α* range for |θ|<0.8 in experiment (a). Further investigation into this interaction from a theoretical standpoint would be valuable, however, we leave this to a future study.

Next, we studied the effect that the hyperparameters had on prediction accuracy across the test and train sets. Here, by test set, we mean all unobserved node-times, and by train set, we mean all observed node-times. Since 80% of the nodes and 80% of the times were observed, this means that $0.8^{2}=64\%$ of all node-times were in the train set, and 36% were in the test set. In this case, the most important parameters were *γ*, *β* and *σ*, with α having a negligible impact on the prediction accuracy.

**Fig 4:**
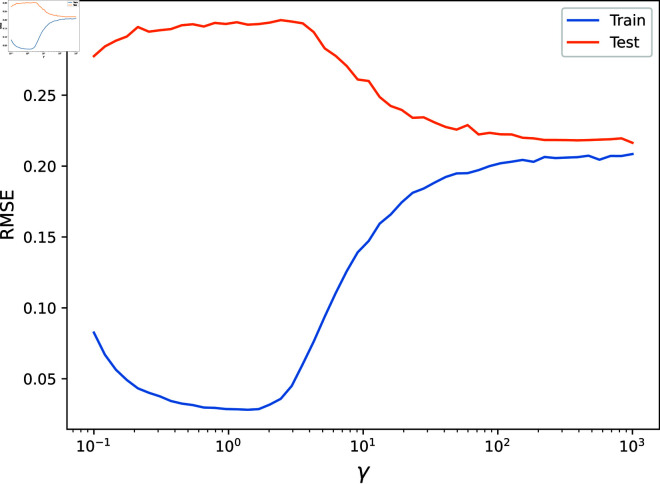
The Root Mean Squared Error on the test and train sets are shown as γ is varied.

**Fig 5:**
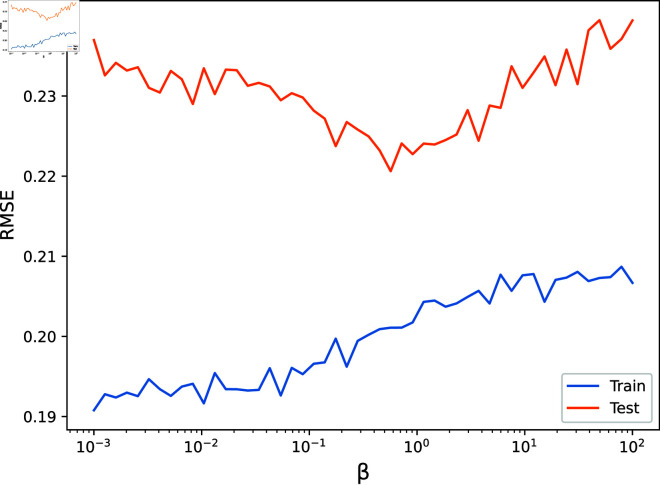
The Root Mean Squared Error on the test and train sets are shown as β is varied.

**Fig 6:**
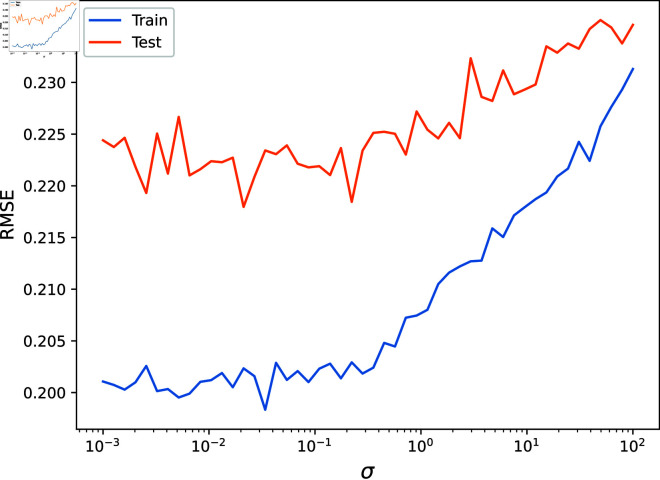
The Root Mean Squared Error on the test and train sets are shown as σ is varied.

**Fig 7:**
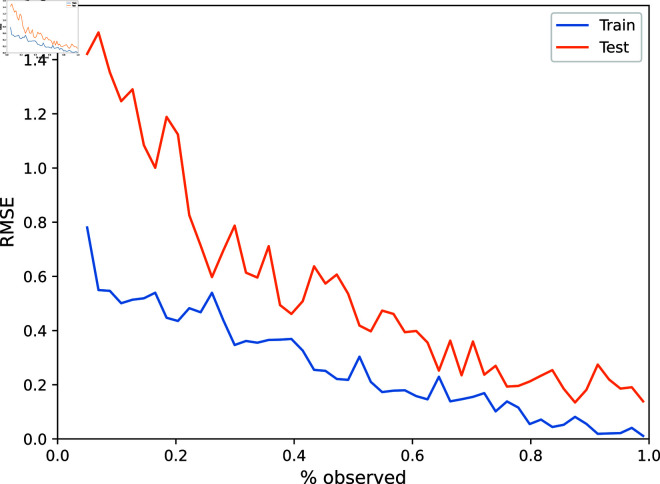
The Root Mean Squared Error on the test and train sets are shown as a function of the total percentage of node-times that were observed.

First, we used gradient descent to find the optimal parameters, with an objective function that evaluated the mean test set RMSE across 50 random realisations of the input data. The optimal values, in this case, were found to be γ=287, β=0.635 and σ=0.263. Next, we fixed two of these parameters and varied the third across a range of values, measuring the mean test and training set RMSE across 50 random realisations of the input data. The results are shown in Figs 4, 5, 6, 7. As is visible, the hyperparameter with the largest effect, and therefore the most important to set appropriately, is *γ*. This is expected, since it acts as a global regularisation parameter. *β* and *σ* also have a small but noticeable effect.

As a final experiment, we also measured the accuracy across both test and train sets as a function of the percentage of nodes and times that were observed. Here, we ran the experiment as before, with the optimal hyperparameters, but increased the total percentage of node-times that were observed from *p* = 0.05 to *p* = 0.99 in 50 equally spaced increments. On each run, we set $N^{\prime }=N\sqrt{p}$ and $T^{\prime }=T\sqrt{p}$, rounded to the nearest integer.

## 5 Latent signal uncertainty via the Laplace approximation

In this final methodology section, we outline the steps for estimating the uncertainty over the latent signal 𝐅 via the Laplace approximation. In the case of KGR, this is in fact not an approximation, but the exact Gaussian process prior. On the other hand, in the GLS case, it is indeed an approximation as point estimates for the uncertain covariance matrices $\Sigma_{N}$ and $\Sigma_{T}$ are used. Here, we derive the approximate posterior for the GLS case only but note that the exact posterior for simple KGR can be restored by setting $\Sigma_{T}$ and $\Sigma_{N}$ equal to the identity matrix of an appropriate size.

Since the latent signal 𝐅 has size N×T, the true covariance matrix specifying its uncertainty has size *NT* × *NT*. Deriving a mathematical expression for this matrix is simple, however, it is generally impractical to compute and store in memory for all but the smallest of problems. For this reason, we take steps to derive an efficient expression for the N×T matrix representing the marginal variance of each element of 𝐅, which is generally the most useful information and allows scaling to relatively large problems.

Consider the negative log-likelihood given in Eq (24). Applying Laplace’s approximation gives the covariance matrix for the uncertainty over the vectorized latent signal.

$$\Sigma_{F}=[-\frac{\partial^{2}\ln\pi (𝐅|𝐘,𝐗)}{\partial \text{vec}(𝐅{)}_{i}\partial \text{vec}(𝐅{)}_{j}}{|}_{𝐅={𝐅}^{⋆}}{]}^{-1}$$ (37)

$$=({𝐒}_{T}^{⊤}\Sigma_{T}^{-1}{𝐒}_{T}⊗{𝐒}_{N}^{⊤}\Sigma_{N}^{-1}{𝐒}_{N}+\gamma {𝐊}^{-1}⊗{𝐇}^{-2}{)}^{-1}$$ (38)

Holding this dense matrix in memory is often impractical, and inverting it is generally out of the question. Instead, we introduce the matrix $\Omega_{F}\in {\mathbb{R}}^{N\times T}$ defined by $\Omega_{F}=\text{mat}({\text{diag}}^{-1}(\Sigma_{F}))$. That is, $\Omega_{F}$ is the diagonal of $\Sigma_{F}$ of length *NT* stacked into an N×T matrix of marginal variances, such that element (*n*,*t*) is the square uncertainty for the *n*-th node at time *t*. Here we present an expression for $\Omega_{F}$ followed by a short derivation.

$$\Omega_{F}=({\tilde{𝐔}}^{-⊤}\circ ({𝐇}^{2}\tilde{𝐔}))\;𝐉\;({\tilde{𝐕}}^{-1}\circ ({\tilde{𝐕}}^{⊤}𝐊))$$ (39)

where ∘ is the Hadamard product,


$${𝐒}_{T}^{⊤}\Sigma_{T}^{-1}{𝐒}_{T}𝐊=\tilde{𝐕}{\tilde{\Lambda }}_{K}{\tilde{𝐕}}^{-1},\;{𝐒}_{N}^{⊤}\Sigma_{N}^{-1}{𝐒}_{N}{𝐇}^{2}=\tilde{𝐔}{\tilde{\Lambda }}_{H}{\tilde{𝐔}}^{-1}$$

and


$${𝐉}_{ij}=\frac{1}{\gamma +{\tilde{\lambda }}_{i}^{K}{\tilde{\lambda }}_{j}^{H}}$$

Note that the eigendecompositions are no longer necessarily performed on symmetric matrices. This means that while $\bar{𝐔}$ and $\bar{𝐕}$ cannot be assumed to be unitary, the eigenvalues are still guaranteed to be positive and real [47]. To prove this expression, first note that $\Sigma_{F}$ can be factorized into the following:


$$\Sigma_{F}=(𝐊⊗{𝐇}^{2})({𝐒}_{T}^{⊤}\Sigma_{T}^{-1}{𝐒}_{T}𝐊⊗{𝐒}_{N}^{⊤}\Sigma_{N}^{-1}{𝐒}_{N}{𝐇}^{2}+\gamma {𝐈}_{T}⊗{𝐈}_{N}{)}^{-1}$$

From here, substitute in the eigendecomposition definitions.


$$\Sigma_{F}=(𝐊\tilde{𝐕}⊗{𝐇}^{2}\tilde{𝐔})\;\text{vec}(\text{diag}(𝐉))\;({\tilde{𝐕}}^{-1}⊗{\tilde{𝐔}}^{-1})$$

This can be split into the following sum.


$$\Sigma_{F}=\sum_{t=1}^{T}𝐊\tilde{𝐕}\delta_{t}{\tilde{𝐕}}^{-1}⊗{𝐇}^{2}\tilde{𝐔}\;\text{diag}({𝐉}_{t})\;{\tilde{𝐔}}^{-1}$$

where $\delta_{t}$ is defined to be a T×T matrix of zeros with element (*t*,*t*) equal to one. From this, it can be seen that the re-stacked diagonal $\Omega_{F}$ is given by the following outer product sum.


$$\Omega_{F}=\sum_{t=1}^{T}{𝐛}_{t}{𝐚}_{t}^{⊤}$$

where


$${𝐚}_{t}=({\tilde{𝐕}}^{-1}{)}_{t}\circ (𝐊\tilde{𝐕}{)}_{t}\;{𝐛}_{t}=({\tilde{𝐔}}^{-⊤}\circ ({𝐇}^{2}\tilde{𝐔}))\;{𝐉}_{t},$$

Or more compactly


$$\Omega_{F}=𝐁{𝐀}^{⊤}=({\tilde{𝐔}}^{-⊤}\circ ({𝐇}^{2}\tilde{𝐔}))\;𝐉\;({\tilde{𝐕}}^{-1}\circ ({\tilde{𝐕}}^{⊤}𝐊))$$

Further detail concerning this derivation can be found in the supplementary materials.

## 6 Experimental results

### 6.1 Spatio-temporal pollutant analysis

In this section, we consider the problem of predicting the concentration of various airborne pollutants, measured across a network of N=1,382 air quality monitoring stations in and around California. Each pollutant type is measured at a unique set of locations and is therefore treated as an independent graph regression task. The goal is to make accurate predictions using weather and environmental features from the previous day. The methods of Kernel Graph Regression and AR(1) GLS Kernel Graph Regression are analysed and compared to some standard baseline algorithms.

Pollutant concentration data was taken from the US Environmental Protection Agency’s air quality monitoring program [59]. Specifically, daily measurements of Ozone, Carbon Monoxide (CO), Nitrogen Dioxide (NO_2_), PM_2.5_ and PM_10_ were taken from January 2017 to April 2021, giving a total of *T* = 1570 days. This data set also contains daily measurements of humidity, pressure, wind speed and temperature at various locations, which we use as additional explanatory variables. In addition, data concerning historical wildfires in California was sourced from the Department of Forestry and Fire Protection in California [60].

### 6.2 Graph construction

A key decision that can significantly impact the effectiveness of a graph signal processing method is how to construct the underlying graph. In certain applications, such as social networks, a sparse graph may be self-evident or relatively simple to construct. However, for a large portion of practical problems, including the current case of geographically placed sensors, it is required to either propose a sensible construction method or learn a graph from available signal data. In this paper we opt for the former, and omit a full discussion of the available techniques, which can be found in, for example, [61].

The graph construction method we use makes use of both pairwise geodesic distances between monitors and the intermediate elevation profile. This is important as environmental processes can be strongly influenced by topography, especially in mountainous regions. Elevation data is sourced from the GLOBE30 project [62]. This dataset gives the approximate height above sea level over a 30 arc-second latitude/longitude grid. While more complex models incorporating land use, prevailing wind direction etc. are possible, we opt for a simpler model for the sake of brevity.

**Fig 8:**
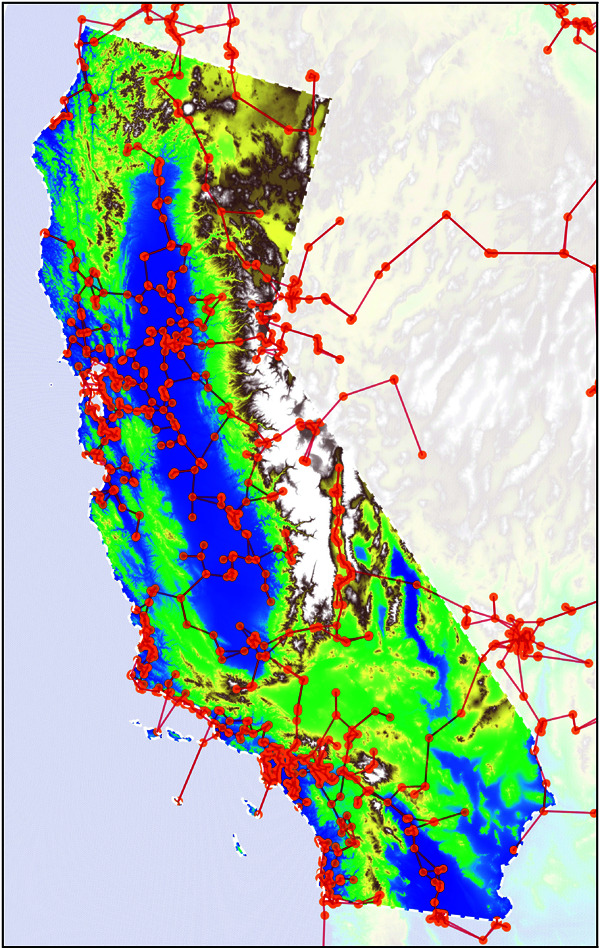
A graphical depiction of the graph connecting the 1382 available monitoring stations, with the map colour representing the elevation profile. This is the graph used in all further experiments. “Reprinted from [38] under a CC BY license, with permission from [Heriott-Watt University], original copyright [2024].” Source available at: http://hdl.handle.net/10399/5041

Our first step is to create an N×N symmetric distance matrix 𝐃. We define the “distance" between two monitors ${𝐃}_{ij}$ to be a weighted combination of their geodesic distance and the vertical relief along the intermediate path. This introduces a hyperparameter defining the relative importance of each component which we learn later via cross-validation. We then use the perturbed minimum spanning tree algorithm outlined in [63] to construct a sparse, fully connected graph. A representation can be found in Fig 8.

### 6.3 Data pre-processing

Several steps were taken to pre-process the raw sensor data before feeding it into the models. Firstly, any stations with more than a total of 15 null readings due to equipment failure, over the three-year period, were discarded. Any remaining missing values were interpolated linearly between the available readings. Secondly, meter readings for each of the pollutant variables, which generally have units of mass or particle count per unit volume, were transformed via log(1+x). This rectified the left skew of the readings, generally producing more symmetric aggregate histograms. The same transformation was also applied to wind speed, however pressure, humidity and temperature, which already had largely symmetric data distributions, were not transformed in this way.

To construct a daily set of features for wildfires, a historical list of all recorded wildfire events in California was used. This dataset includes information about the start and end date of each fire, along with the central location and the total ground area burned. For each fire we assumed that the burn rate rose and fell with a Gaussian shape, peaking at the midpoint of the burn window, such that 95% of the area burns within the specified time range. From this we estimate the total area burning on any given day in eleven different regions in California.

The final step before estimation was to transform all variables so that they were scaled and translated to achieve a unit marginal variance and zero mean. PCA dimensionality reduction was then performed on each of the feature groups individually keeping enough dimensions to preserve 90% of the total variance for that group. The resultant input data for 𝐗 and 𝐘 had 1570 columns representing the dates from 2017-01-02 to 2021-04-20. Each of the *N* rows of 𝐘 held a time series of readings for a unique monitoring sensor with a well-defined location. The columns of 𝐗 contain first the PCA components of the weather conditions, second the approximate number of acres burning in each of the eleven regions. Regression was then performed for each of the five different pollutants. Table 3 gives information about the total number of features that were abvailable after perfroming these processing steps.

**Table 3:** Information about the number of available features before and after PCA.

	Before PCA	% active	After PCA
Ozone	165	11.9	12
CO	63	4.6	8
NO_2_	90	6.5	11
PM2.5	71	5.1	10
PM10	86	6.2	10
Wind	136	9.8	11
Pressure	46	3.3	7
Temperature	146	10.6	12
Humidity	82	5.9	9
Fire	11	N/A	11

### 6.4 Results

To test the performance of the KGR and GLS KGR methods, we compared them against several other regression algorithms, namely Ordinary Least Squares (OLS) regression, Ridge regression, Lasso regression and Elastic Net regression.

**Fig 9:**
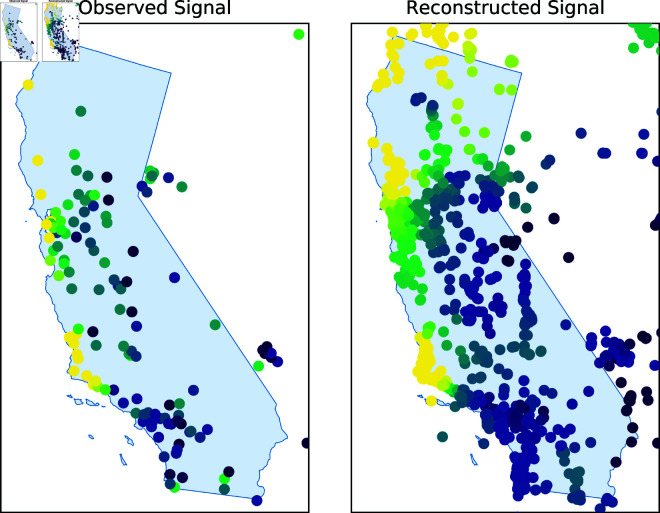
Left: the observed graph signal for Ozone on a particular day represented via a colour map. Right: prediction for the reconstructed latent graph signal across the entire network on the same day made by the GLS KGR method. “Reprinted from [38] under a CC BY license, with permission from [Heriott-Watt University], original copyright [2024].” Source available at: http://hdl.handle.net/10399/5041

**Fig 10:**
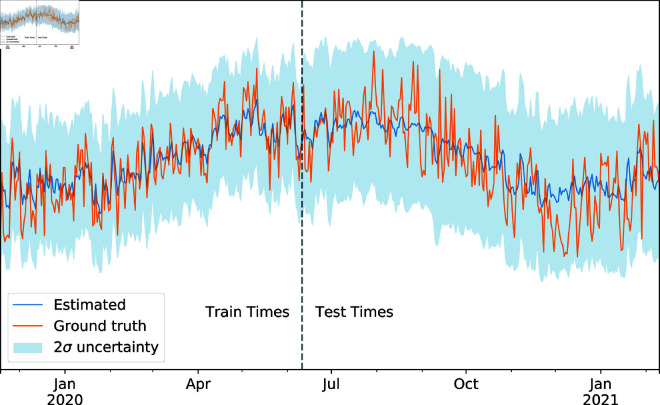
A section of the Ozone time-series signal predicted by GLS KGR at a particular unobserved node is shown along with the ground truth. The blue shading depicts two standard deviations of prediction error arising from latent signal uncertainty as calculated by the Laplace approximation.

For each model tested, all relevant hyperparameters were tuned using cross-validation. First, the input data was split such that the first 80% of the days served as a training/validation set and the final 20% of the days as a test set. The train/validation set was then split into four folds of 219 days each uniformly at random. Hyperparameters were set by attempting to minimise the mean squared error averaged across each of these four folds, using three to train and one to validate accordingly. Final results were then reported on the test set. Fig 9 shows the predicted Ozone levels across the full graph (right) on a particular day given the partially observed signal (left). As expected, the output is fairly smooth across the network, indicating that the model has successfully utilised information from the graph structure. Fig 10 shows the output from the GLS KGR algorithm at a particular unobserved node. As is visible, the estimated reading approximates the ground truth well, especially during train times. In addition, the uncertainty about the estimate notably increased during the test times, further indicating a healthy model output.

Table 4 shows the mean squared error as reported on the test set after hyperparameter tuning. As is visible, either GLS KGR or KGR performs the best by this metric across all pollutants. Table 5 shows the total compute time for each method on a 4-core i7 intel CPU.

**Table 4:** Mean squared Error on all unseen data.

	Ozone	CO	NO_2_	PM2.5	PM10
GLS KGR	0.6444	0.8207	0.6912	0.7512	0.6899
KGR	0.6539	0.8438	0.7201	0.7809	0.6728
Ridge	0.7298	0.9704	0.7316	0.7819	0.7195
Lasso	0.7283	0.9881	0.7296	0.7763	0.7222
Elastic Net	0.7201	0.9662	0.7254	0.7816	0.7181
OLS	1.1276	2.4638	1.0311	1.1715	1.1784

**Table 5:** The run time for each algorithm in seconds.

	Ozone	CO	NO2	PM2.5	PM10
GLS KGR	5.483	8.645	5.996	6.726	5.945
KGR	0.808	0.914	0.898	0.834	0.767
Ridge	1.156	1.161	1.091	1.070	1.040
Lasso	2.143	2.089	2.153	1.874	2.076
Elastic Net	2.030	2.139	2.501	2.071	2.224
OLS	1.520	1.112	1.305	1.056	1.225

## 7 Discussion

### 7.1 Hyperparameter tuning

One weakness with KGR and GLS KGR in practice is that several hyperparameters must be tuned to produce an accurate model, namely α,β,γ and σ, as well as any used in the graph construction period. Each is important and can have a significant effect on the output produced. For example, insufficient regularisation via γ can cause severe over-fitting and result in unduly small entries within the estimated cross-covariance matrix $\Sigma_{N}$. Similarly, setting α too high can effectively drive the estimated value of θ to zero, while setting it too low can result in singular matrices if the data is at all non-stationary. This creates a large, non-convex search space that can be both costly to explore and full of local minima. The degree to which this is an issue depends largely on the size of the problem at hand. For relatively small problems, such as the one considered here with ~1000 nodes and ~1000 time steps, well-known scalar minimization algorithms such as Nelder-Mead can help automate this process. However, with significantly larger problems hyperparameters would have to be carefully selected based on domain knowledge.

### 7.2 The normalised graph Laplacian

One model choice not discussed so far is the question of whether to use the normalised or un-normalised version of the graph Laplacian. Until now, we have assumed the regular Laplacian, 𝐋, is being used, however, the normalised version, defined by $\tilde{𝐋}={𝐃}^{-1/2}𝐋{𝐃}^{-1/2}$, has properties that may make it preferable in some circumstances. For example, graph penalties constructed using $\tilde{𝐋}$ give equal weight to all nodes, whereas penalties constructed using 𝐋 implicitly favour high-degree nodes because they appear more frequently in the sum of Eq (1) [27]. This may or may not be desirable depending on the problem at hand. Another potential benefit of $\tilde{𝐋}$ is that its eigenvalues are guaranteed to fall in the interval [0,2] which makes the graph-regularisation parameter β easier to set and interpret, as well as making a sensible comparison across problems possible. In the case of environmental monitoring networks, our initial experiments indicate that the normalized Laplacian may result in slightly better performance, however, further investigation is necessary.

### 7.3 Options for increasing scalability

In the previous analysis, we assumed that, while the graph Laplacian 𝐋 is often sparse, the filter and kernel matrices 𝐇 and 𝐊 were dense. This typically means that the primary bottleneck for KGR and GLS KGR is the eigendecomposition of these matrices, even in their down-sampled form. For large problems, it may be sufficient to only calculate the first *k* eigenvectors and eigenvalues, which can be performed efficiently for sparse matrices. Therefore finding sparse representations for 𝐇 and 𝐊 can be highly beneficial for large applications. Numerous methods exist in the literature on kernel regression for improving computational robustness. One, in particular, is the Nystrom method, which uses a low-rank approximation to the kernel matrix [64]. This can reduce the computational complexity of solving the linear system to $O(M^{3}+T^{2}M)$, and the memory requirements to *O*(*M*^2^ + *MN*), where *M* is the chosen number of data examples.

One way to create a sparse graph filter is to simply use a low-degree polynomial for the filter function η(λ). In this way, 𝐇 can be calculated efficiently as η(𝐋) and decomposed faster. When it comes to the kernel matrix, a large literature also exists on compactly supported kernels, for example, the Wendland kernel [65]. Here, an integral operator is applied recursively to Askey’s truncated power functions to create a set of compactly supported radial basis functions, which are guaranteed to be smooth, continuous and positive definite. Fast algorithms for the computation of these basis functions have been outlined in [66].

## 8 Conclusions and future work

This paper has contributed a statistical analysis of regression methods for signals defined over networks. Drawing upon recent work on Kernel Graph Regression, Gaussian Processes over Graphs and graph signal reconstruction, we proposed the method of GLS KGR and demonstrated its effectiveness on a relevant task. In particular, we addressed the situation where a partial observation of an *N*-node graph signal is made at a subset of *T* time-points, deriving the steps for an AR(1) autocorrelation regression model, with general cross-correlation. By assuming a matrix-normal error distribution, an algorithm was designed with *O*(*N*^3^  +  *T*^3^) complexity at each iteration. Finally, the Laplace approximation was used to derive a lower bound for the marginal prediction error arising from latent signal uncertainty. This was tested and shown to be effective on real data taken from a network of pollutant monitoring stations.

One assumption in this work was that the unobserved nodes remain so over the lifetime of the problem. This is somewhat unrealistic in applications such as sensor networks where equipment may temporarily fail. In the future, it would be valuable to work on adapting these algorithms for arbitrary missing values. Another worthwhile investigation would be a systematic study into the effect of different graph construction methods within the context of graph regression.
